# Comprehensive analysis of microRNA and metabolic profiles in bovine seminal plasma of different semen quality

**DOI:** 10.3389/fvets.2023.1088148

**Published:** 2023-03-28

**Authors:** Wei Cao, Wenqiang Sun, Shiyi Chen, Xianbo Jia, Jie Wang, Songjia Lai

**Affiliations:** ^1^Key Laboratory of Livestock and Poultry Multi-omics, Ministry of Agriculture and Rural Affairs, College of Animal Science and Technology (Institute of Animal Genetics and Breeding), Sichuan Agricultural University, Chengdu, China; ^2^Farm Animal Genetic Resources Exploration and Innovation Key Laboratory of Sichuan Province, Sichuan Agricultural University, Chengdu, China; ^3^College of Animal Science and Technology, Sichuan Province General Station of Animal Husbandry, Chengdu, China

**Keywords:** bovine seminal plasma, semen quality, miRNAs, metabolites, high-throughput sequencing

## Abstract

**Background:**

Seminal plasma plays a pivotal role in modulating sperm viability and function. However, the underlying mechanisms have not been fully elucidated.

**Method:**

In this study, the bull semen production records of core breeding farms and bull stations in the past 10 years were analyzed.

**Results:**

We found that the semen of 5-year-old bulls collected for the first time is of the best quality (*p* < 0.05). Despite the bull semen collected under the above conditions, low-quality sperm is still obtained from part of bulls due to individual differences. Interestingly, seminal plasma from normal semen is capable of improving low-quality semen motility. To identify the potential key factors in seminal plasma, the differences in miRNA and metabolite profiles between normal and low-quality seminal plasma were analyzed. We found that 59 miRNAs were differently expressed, including 38 up-regulated and 21 down-regulated miRNAs. Three hundred and ninety-one and 327 significantly different metabolites were identified from the positive and negative ion models, respectively. These multiple miRNAs and metabolites collectively contribute to the motility of sperm, subsequently, affect semen quality.

**Discussion:**

Together, these results not only revealed the critical factors of seminal plasma improving sperm quality but also provided potential miRNA- or metabolite-based biomarkers to identify the high semen quality.

## Introduction

Artificial insemination has become routine reproductive biotechnology in modern livestock production systems (1–3). Semen quality is one of the most important indicators used for the evaluation of reproductive efficiency. A plethora of factors are capable of affecting semen quality, subsequently, leading to decrease male fertility (4). Among them, some factors, such as age and collection frequency, can be intervened and managed for better semen quality. Thus, it is worthwhile to investigate the effect of age and collection frequency influencing semen quality.

During ejaculation, spermatozoa are mixed with a fluid medium called seminal plasma, which serves as a nutritive–protective medium for spermatozoa and mediates the chemical function of the ejaculate (5, 6). Biochemical analyses indicate that seminal plasma consists of proteins, enzymes, minerals, lipids, hormones, cytokines, and a series of metabolites (6–8). Accumulating evidence shows the composition and content of metabolites in seminal plasma are closely related to energy production, motility, protection, fertilization, and metabolism of sperm (9–11). Importantly, recent literature suggests an important role of seminal plasma metabolites in interaction with complementary changes in gene expression (3). MicroRNAs (miRNAs), a class of short non-coding RNAs consisting of ~ 22 nucleotides each, are able to regulate gene expression by binding to their complementary sites within the untranslated regions of target mRNAs (12). Meanwhile, miRNAs are known to participate in the regulation of various biological processes and have within the past decade been identified as key regulators in the control of metabolic homeostasis (13–15). To date, multiple experiments have been designed to combine transcription and metabolite results using correlation and clustering analyses as well as illustrating their connections in networks (16–19), which provide new insights into the understanding of the molecular mechanism.

During the past years, the profiles of seminal plasma miRNAs and metabolites have been analyzed independently (7, 10, 20, 21), meaning that limited information can be directly retrieved from the current literature about the correlations of miRNAs and metabolites in seminal plasma. In the present study, to explore the regulatory networks in the seminal plasma at the level of the miRNA and metabolome, we identified and reported the differences in the miRNA and metabolite profiles between normal and low-quality semen seminal plasma. In addition, functional analyses were performed to understand the enriched pathways of the significantly changed miRNAs and metabolites.

## Materials and methods

### Semen quality data records

Semen quality data records were recorded from 2008 to 2018 of 854 Simmental bulls maintained at Yangping Breeding Cattle Farm and Sichuan Bull Breeding Station in Sichuan Province, China. Information of records includes age at first semen collection, age at first frozen semen, semen production period, frozen semen production period, last collected semen age, last frozen semen age, culling age, and production record (sperm motility, sperm density, and semen production).

### Samples and seminal plasma preparation

All bulls (Chinese Simmental cattle) were aged 5 years and were raised on a farm in the Animal Husbandry Station of Sichuan Province under the same management conditions and received the same nutrition. The bulls were under regular examination and free of infectious diseases (BHV1, BVDV, brucellosis, bovine leucosis, and et al.). During last year, freshly ejaculated sperm were collected from individuals using an artificial vagina, and sperm motility was evaluated every time for each bull using computer-aided sperm analyzer (CASA; CEROS, IMV Technologies, France) systems by a specific lab technician immediately after collection. According to the World Health Organization guidelines and national standards (GB 4143-2008, China), semen which ≥65% semen motility, ≥85% morphologically normal sperm and, ≥6 × 10^8^ semen density per mini liter was set as normal semen, while the semen which ≤ 65% semen motility, ≤ 85% morphologically normal sperm or ≤ 6 × 10^8^ semen density per mini liter was set as poor-quality. Based on the sperm motility, morphologically normal sperm, and semen density records, four normal and four low-quality semen were selected for sperm collection and seminal plasma isolation in the present study. Seminal plasma was separated from sperm at room temperature within 2 h of sampling using a two-step centrifugation method: the supernatant was centrifuged first at 800 g for 8 min at 4°C, and then at 12,000 *g* for 5 min at 4°C until no spermatozoa were observable by microscopy. After the second centrifugation, seminal plasma was transferred to a 2 mL microcentrifuge tube and immediately subjected to total RNA isolation.

### Library construction and sequencing of small RNA

Small RNAs were extracted from seminal plasma using the miRNeasy serum/plasma kit (Qiagen, Valencia, CA, USA) according to the manufacturer's instructions. The quality was examined by the NanoDrop 2000 Spectrophotometer (Thermo Fisher Scientific, Wilmington, DE, USA) after the integrity was determined by RNA Nano 6000 Assay Kit of the Agilent Bioanalyzer 2100 System. After that, sequencing libraries were generated using NEB Next Ultra small RNA Sample Library Prep Kit for Illumina (NEB, Ipswich, MA, USA), following the manufacturer's protocol. The purified RNA was ligated with specific adapters for Illumina processing on an Illumina Hiseq™ 2500 platform and reads were generated.

### miRNA identification and differential expression analysis

Raw data were subjected to stringent quality control measures to remove low-quality reads (phred quality scores ≤ 30), ambiguous reads [reads containing over 10% of poly (*N*)], adaptor sequences, and reads shorter than 18 nucleotides or longer than 30 nucleotides. Filtered sequences (clean reads) were then annotated with Silva, GtRNAdb, Rfam, and Repbase to identify rRNA, tRNA, snRNA, snoRNA, ncRNA, and repeat sequences using Bowtie (22). Subsequently, the remaining unannotated clean reads were aligned against the cattle reference genome (Bos_taurus.ARS_UCD1.2) using Bowtie (22) and the mapped sequences were further analyzed using miRbase (http://www.mirbase.org/), and miRDeep2 (23) to identify miRNA and novel miRNA. Expression levels of miRNAs were calculated by DESeq2 (24), based on the reads per kilobase million (TPM) algorithm determination of different expression levels of miRNAs between the two groups, with the threshold for significant differently expression set as |log2(fold change) |≥1 and *p*-value ≤ 0.01.

### miRNA target prediction and functional annotation

Target gene prediction was implemented using miRanda (25) and targetscan (26). Gene Ontology (GO) and Kyoto Encyclopedia of Genes and Genomes (27) (KEGG) were employed to analyze potential functions and pathways of the differently expressed miRNAs of target genes, and the criterion of significance was *p* < 0.05.

### Metabolite profiling of seminal plasma and data analyses

Metabolite profiling was performed at Biomarker Technologies Co., Ltd. (Beijing). Briefly, an untargeted metabolic profiling method was performed to analyze the relative levels of metabolites of the samples. Metabolite profiling was conducted using ultra-performance liquid chromatography (Waters Corporation, Milford, MA, United States) coupled to quadrupole-time-of-flight mass spectrometry (UPLC/Q-TOF MS), and unbiased metabolic profiling was conducted in positive electrospray ionization (ESI+) and negative electrospray ionization (ESI–) mode. The TripleTOF 6600 mass spectrometry (AB SCIEX, USA) was used for its ability to acquire MS/MS spectra on an information-dependent basis during the experiment. In this mode, the acquisition software (Analyst TF 1.7, AB SCIEX, USA) continuously evaluates the full scan survey MS data as it collects and triggers the acquisition of MS/MS spectra depending on preselected criteria. In each cycle, the most intensive 12 precursor ions with intensity above 100 were chosen for MS/MS at collision energy (CE) of 30 eV. The cycle time was 0.56 s. ESI source conditions were set as follows: Gas 1 as 60 psi, Gas 2 as 60 psi, Curtain Gas as 35 psi, Source Temperature as 600°C, Decluttering potential as 60 V, Ion Spray Voltage Floating (ISVF) as 5,000 or −4,000 V in positive or negative modes, respectively. Then, an in-house MS2 database was applied for metabolite identification. The XCMS program (28) was subsequently used for noise elimination, peak picking, alignment, and retention time correction of the raw data. Peak intensities of metabolites were normalized to the total spectral intensity. An orthogonal partial least-squares discriminant analysis (OPLS-DA) model was then used to observe the differences in metabolic composition between the Normal and low-quality groups, and the corresponding R2X, R2Y, and Q2Y values were calculated for statistical analysis. Together with the VIP value from OPLS-DA and fold change, the metabolites with VIP >1 and |log2(fold change) |≥1 were considered as potential discriminating metabolites. Finally, the KEGG metabolic pathways of the differential metabolites were evaluated using the cluster Profiler package in R software (29). The association analysis between the significantly changed miRNA and metabolites were determined by the |coefficient (CC) value |≥0.8 and coefficient *p*-value (CCP) < 0.05 using the Spearman method.

## Results

### Factors affecting semen quality

**Figure 1:**
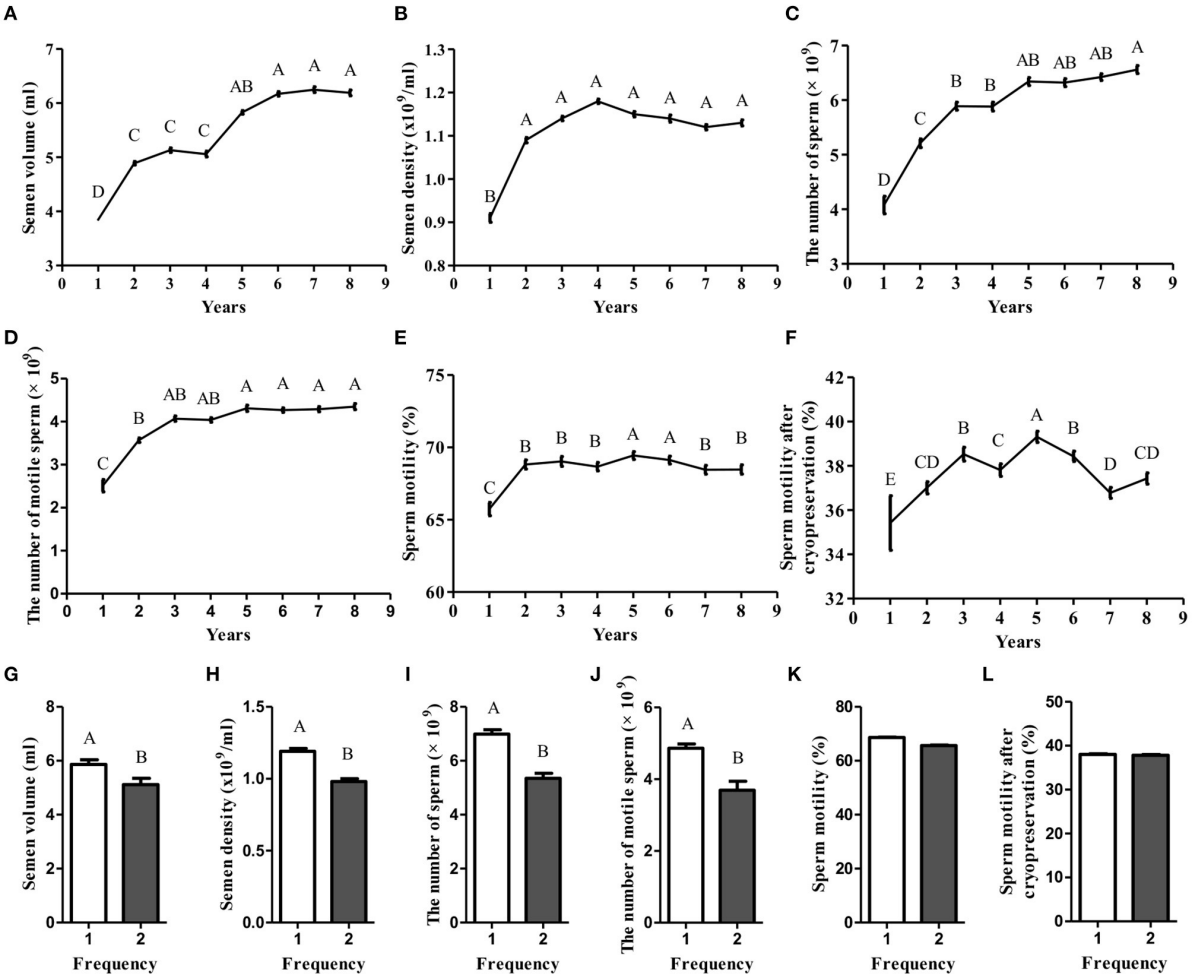
The effect of age and collection frequency affecting semen quality. **(A–F)** The effect of age on semen volume, semen density, the number of sperm, the number of motile sperm, sperm motility, and sperm motility after cryopreservation; **(G–L)** The effect of collection frequency on semen volume, semen density, the number of sperm, the number of motile sperm, sperm motility, and sperm motility after cryopreservation.

A plethora of factors are capable of affecting semen quality, subsequently, leading to decrease male fertility (4). Thus, to identify the key factors, the bull semen production records of core breeding farms and bull stations in the past 10 years were analyzed. We found that as the age increased, the semen volume, semen density, total sperm count in a single ejaculation, and total viable sperm count in a single ejaculation all showed a gradually increasing trend (Figures 1A–D), while fresh sperm motility and thawed sperm motility showed an irregular trend of first increasing and then decreasing (Figures 1E, F). These data suggest that age is one of the key factors affecting semen quality. Moreover, the semen quality of 5-year-old bulls is the best (*p* < 0.05). In addition to age, the effect of semen collection frequency on semen quality was determined. We found that the quality of the semen collected for the first time was significantly better than that of the semen collected the second time (*p* < 0.05; Figures 1G–J). However, the sperm motility and sperm motility after cryopreservation did not show any difference (Figures 1K, L). Together, these data suggest that the semen of 5-year-old bulls collected for the first time is of the best quality.

### Seminal plasma from normal semen improves low-quality semen motility

**Figure 2:**
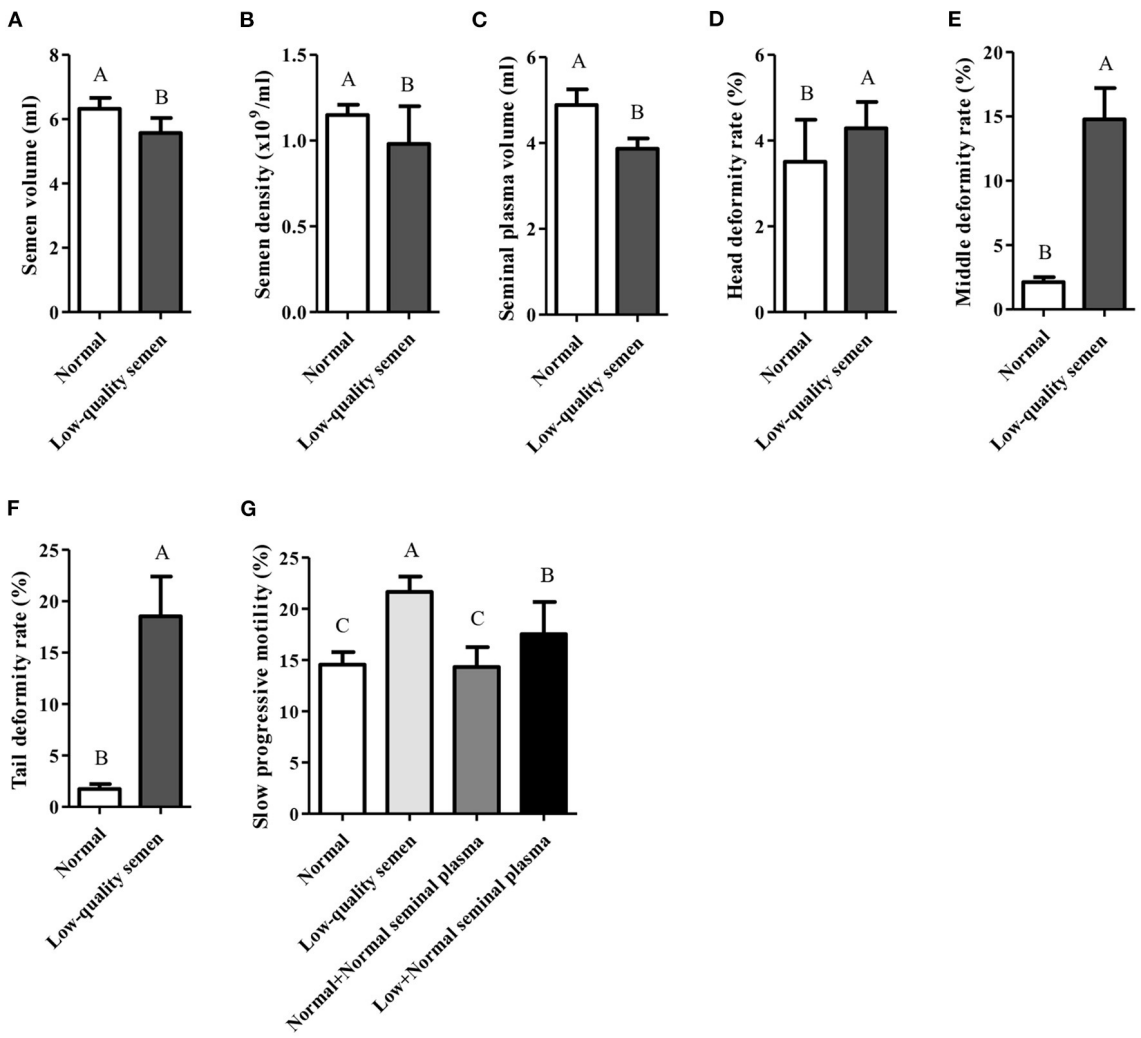
Comprehensive analysis of normal and low-quality semen. **(A–F)** Comprehensive analysis of semen volume, semen density, seminal plasma volume, head deformity rate, middle deformity rate, and tail deformity rate; **(G)** Seminal plasma from normal semen improves low-quality semen quality.

Despite the bull semen can be collected under the above conditions, low-quality sperm is still obtained from part of bulls due to individual differences. According to the records of core breeding farms and bull stations in the past 10 years, only 87 of 845 bulls could consistently produce normal semen. Compared with the normal semen group, the semen collection volume, sperm density and seminal plasma volume of the low-quality semen group were all significantly lower (*p* < 0.05; Figures 2A–C), while the deformity rate of the sperm head, trunk, and tail were all significantly higher (*p* < 0.05; Figures 2D–F). Moreover, the slow progressive motility was also significantly higher in the low-quality semen group (*p* < 0.05; Figure 2G). Seminal plasma is known to play a pivotal role in stimulating and supporting sperm by providing a nutritious and protective environment as well as enhancing sperm motility in the female reproductive tract (7). Interestingly, we found that if the seminal plasma of low-quality semen was replaced by normal seminal plasma, the slow progressive motility will be significantly reduced (Figure 2G), suggesting that the normal seminal plasma can improve low-quality semen motility.

### Seminal plasma miRNA analysis of different semen quality

It has been indicated in numerous studies that abundant miRNAs and metabolites are present in seminal plasma (7, 30). In order to clarify how normal seminal plasma improves low-quality semen motility, miRNA-seq was performed by using normal and low-quality seminal plasma samples. In total, 92,124,043 clean reads were filtered from 92,243,085 raw reads. After mapping the obtained clean reads to the reference genome, other types of RNA and repeated sequences were excluded (Supplementary Table 1). Finally, a total of 2,983 miRNAs, including 727 known miRNAs and 2,256 novel miRNAs, were identified from the samples of the present study. The numbers of known miRNAs and novel miRNAs in each sample ranged from 396 to 620 and 955 to 1,364, respectively (Supplementary Table 2).

**Figure 3:**
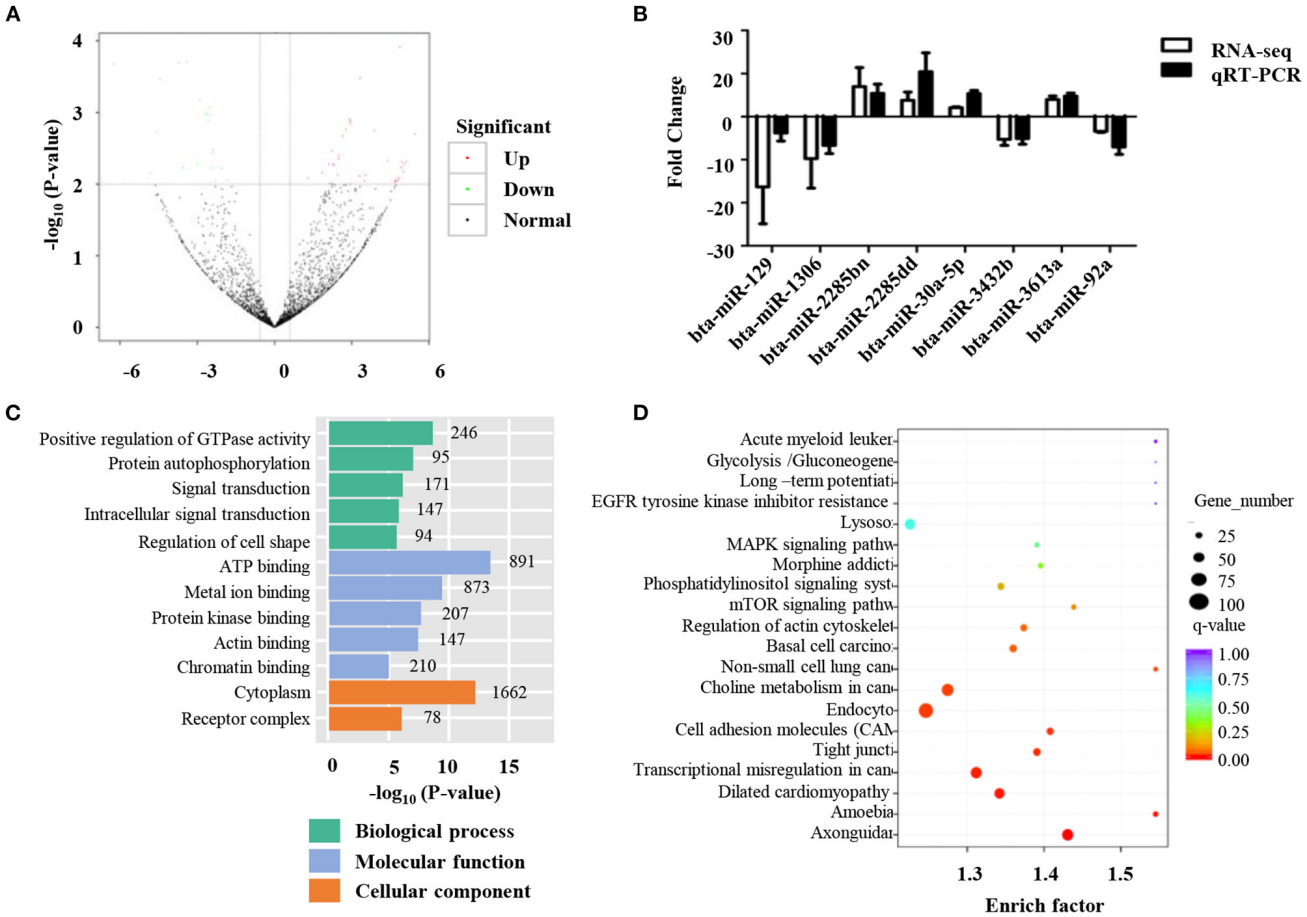
Identification of differentially expressed miRNAs. **(A)** Volcano plots of the differential microRNA between normal and low-quality semen group; **(B)** The qRT-PCR validation of eight differential microRNAs; **(C)** Gene ontology (GO) analysis of the predicted target genes regulated by the differentially ex-pressed miRNAs. **(D)** Kyoto Encyclopedia of Genes and Genomes (KEGG) pathway analysis of the predicted target genes regulated by the differentially expressed miRNAs.

Differentially expressed miRNAs in the normal and low-quality semen groups were differentially analyzed using the TMP method. Fifty-nine miRNAs were found to be differently expressed between the two groups (Figure 3A). Specifically, 38 miRNAs were up-regulated and 21 miRNAs were down-regulated (Figure 3A and Supplementary Table 3). To verify the reliability of these results, qRT-PCR was performed, and the results were consistent with miRNA-seq data (Figure 3B). To determine the biological functions of differentially expressed miRNAs, miRanda, and targetscan software were used to predict the target genes of the differentially expressed 59 miRNAs, as well as GO and KEGG functional enrichment analysis. GO enrichment analysis showed that 336 were enriched in the biological process category, 78 in the cell composition category, and 133 in the molecular function category (Figure 3C and Supplementary Table 4). In the biological process category, the pathways of target genes mainly include positive regulation of transcription from RNA polymerase II promoters, positive regulation of GTPase activity, and negative regulation of transcription from RNA polymerase II promoters (Figure 3C). In the molecular function category, ATP binding, RNA polymerase II core promoter sequence-specific DNA binding, and metal ion binding are the biological processes that are more concentrated in target genes (Figure 3C). The KEGG results showed that 59 differentially expressed miRNAs were significantly enriched in 55 KEGG pathways (Figure 3D and Supplementary Table 5). Pathways with higher enrichment include Axon guidance, Tight junction, and cell adhesion molecules. KEGG pathway analysis of the target genes of the up-regulated and down-regulated miRNAs showed that the target genes of the up-regulated miRNAs were mainly related to axon guidance, basal cell carcinoma, and cell adhesion molecule signaling pathways (Figure 3D), while the down-regulated miRNAs focus on the MAPK signaling pathway and axon guidance signaling pathway.

### Seminal plasma metabolomics analysis of different semen quality

**Figure 4:**
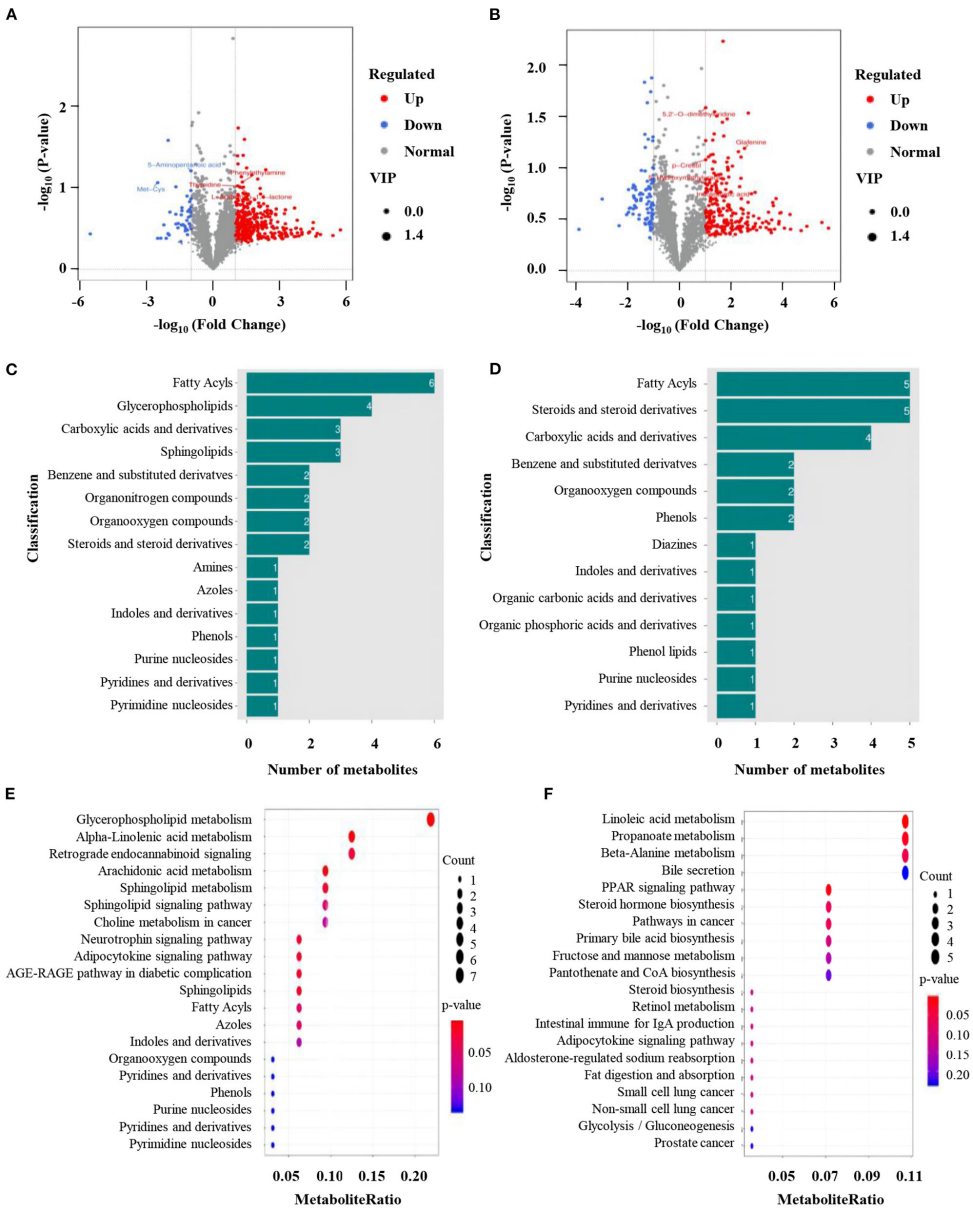
Identification of differentially expressed metabolites. **(A, B)** Volcano plots of the differential metabolites between normal and low-quality semen group; **(C, D)** Gene ontology (GO) analysis of significantly changed metabolites from positive and negative model. **(E, F)** Kyoto Encyclopedia of Genes and Genomes (KEGG) pathway analysis of significantly changed metabolites from positive and negative model.

To further clarify how normal seminal plasma improves low-quality semen quality, the differences in metabolites between normal and low-quality groups were determined. OPLS-DA was first conducted to identify the differential metabolites between the two groups. All Q2 values (ESI+: Q2 = −0.0477 or ESI–: Q2 = −0.607) in blue on the left were lower than the original points in green on the right, indicating the mode reflect the real situation without over-fitting. Briefly, 3,391 and 2,892 metabolite signals from positive and negative ion modes, respectively (Supplementary Table 6). Three hundred and ninety-one and 327 significantly different metabolites were identified from the positive and negative ion models, respectively (Figures 4A, B). Among them, 347 metabolites were up-regulated and 44 metabolites were down-regulated in positive ion mode (Figure 4A). The numbers of up-and down-regulated metabolites in negative ion mode were 229 and 88 (Figure 4B). In the positive ion mode, the greatest difference in the differential metabolites was the abundance of 6 fatty acids in fatty acids, followed by glycerophospholipids, carboxylic acids and their derivatives, and sphingolipids (Figure 4C). The composition of differential metabolites in seminal plasma with high sperm motility in negative ion mode was similar to that in positive ion mode, with the most differences being fatty acids and sterols and their derivatives, followed by carboxylic acids and their derivatives (Figure 4D). KEGG enrichment analysis was used to functionally annotate the significantly changed metabolites, and the results showed that the metabolic pathways with the highest enrichment in positive ion mode were glycerophospholipid metabolism, α-linolenic acid, and arachidonic acid metabolism pathways. The differential KEGG enrichment pathways in the negative ion mode were metabolic pathways such as linoleic acid metabolism and propionic acid metabolism (Figures 4E, F).

## Discussion

Semen quality is one of the most important indicators for the evaluation of reproductive efficiency. The semen quality is affected by many factors, such as age, and collection frequency (4). Our data showed that as the bull's age increased, the semen volume, fresh precision, total sperm count in a single ejaculation, and total viable sperm count in a single ejaculation all showed a gradually increasing trend, while fresh sperm motility and thawed sperm motility showed an irregular trend of first increasing and then decreasing. Moreover, the semen of 5-year-old bulls collected for the first time is of the best quality. These findings are consistent with a previous report (31). Despite the bull semen can be collected under the right conditions, low-quality sperm is still obtained from part of bulls due to individual differences. Interestingly, if the seminal plasma of low-quality semen was replaced by normal seminal plasma, the slow progressive motility will be significantly reduced, indicating that the normal seminal plasma can improve low-quality semen quality.

It has been acknowledged that semen quality corresponds to the structural and functional integrity of sperm and ultimately influences the animal's fertility (32, 33). The regulation of sperm semen consists of a series of complicated biological processes that are greatly influenced by seminal plasma (7, 34, 35). In mammals, seminal plasma mainly comes from secretions of the epididymis and accessory sex glands (2). It has been indicated in numerous studies that abundant miRNAs and metabolites are present in seminal plasma and are closely related to the functions of semen and breeding efficiency (7, 30). Using high-throughput technologies, we conducted a comprehensive and systemic investigation of the miRNA and metabolite profiles in seminal plasma samples with high and low motility. As a result, a large amount of information, including differently expressed miRNAs, differently changed metabolites, significantly enriched pathways, and numbers of putative association networks between miRNAs and metabolites were identified in the present study. Interestingly, part of miRNA has been proven to be involved in spermatogenesis. For example, miR-92a is linked to the fertilizing potential of bovine sperm and is crucial for Sc-mediated induction of active spermatogenesis at puberty and regulation of male fertility (36, 37). MiR-1306 was differently expressed in crossbred bull semen (38). MiR-30a-5p is overexpressed in non-obstructive azoospermia (NOA) patients compared to obstructive azoospermia (OA), and miR-30a-5p could potentially target the KDM3A transcript and hinder it from being translated. As a result, the downstream genes TNP1, PRM1, and PRM2 remain silent. All of these factors combine to induce male infertility in men with NOA (39). MiR-126 can stimulate cell proliferation and restrain the apoptosis of immature porcine Sertoli cells by targeting the PIK3R2 gene. Through this process, miR-126 further activates the PI3K/AKT signaling pathway. miR-126, PIK3R2, and the PI3K/AKT signaling pathway might play pivotal regulatory roles in porcine spermatogenesis by deciding the destiny of immature Sertoli cells (40).

The sperm quality is highly dependent on multiple signaling mechanisms and metabolic pathways because sperm is a kind of transcriptionally silent cell (33). Axon guidance was the most enriched KEGG pathway identified in this work. Previous studies mainly concentrated on the mechanisms of axon guidance in the nervous system, while recent works have reported that axon guidance also acts non-canonically to regulate cell migration and gene expression, suggesting that the axon guidance pathway may have the ability to control the motility of sperm *via* underlying molecular signaling (41, 42). Simultaneously, the predicted target genes of the differently expressed miRNAs were highly grouped in the tight junction pathway. Tight junctions participate in forming the blood-testis barrier in the mammalian testes and perform important roles in the migration of germ cells (43, 44). Meanwhile, the junctional adhesion molecule (JAM)-A has been reported to be involved in sperm tail formation and is essential for normal motility (45). These reports collectively indicate that miRNA is capable of regulating sperm motility through potential targets in the tight junction pathway. The mitogen-activated protein kinase (MAPK) signaling pathway consists of three subfamilies named c-Jun N-terminal kinase (JUK), extracellular signal-regulated kinase (ERK), and p38. Of these, ERK and p38 are widely known to be implicated in sperm motility, although different effects of ERK 1/2 have been observed in the regulation of sperm motility (46, 47). On the other hand, Rho GTPase activating protein 6 (ARHGAP6), a substrate of ERK 1/2, is a Rho GTPase-activating protein (48). It is known that most axon guidance receptors have the ability to regulate Rho-family small GTPases *via* cytoplasmic proteins (49). This evidence also suggests the involvement of axon guidance pathway in the regulation of sperm motility. Additionally, the JUN and p38 MAPK signaling pathways also regulate the dynamic of the tight junction. Taken together, the axon guidance, tight junction, and MAPK signaling pathways probably combine with one another to mediate sperm motility. Energy-related pathways are the basic requirements for sperm motility. The results of the present study showed that a total of 845 target genes were grouped into the GO pathway analysis group of ATP binding. In the enriched pathways of metabolites, many lipid metabolism pathways were also found to be significantly grouped. During the glycolysis process, glucose is converted into pyruvate, with energy being released in the form of ATP and NADH (33, 50). Similarly, the process of lipid metabolism is also an important source of energy (51). Therefore, low efficiency of glucose and lipid metabolism leads to energy shortage and consequently decreases the motility of sperm.

## Conclusion

In conclusion, we found that the semen of 5-year-old bulls collected for the first time is of the best quality. Importantly, seminal plasma from normal semen improves low-quality semen quality. The differences of miRNA expression and metabolite profiles between normal and low-quality revealed that multiple genes, metabolites, and pathways collectively contribute to the motility of sperm. These results have potential application for the further development of miRNA- or metabolite-based markers, as well as provide abundant data for the studies in the future.

## Data availability statement

The datasets presented in this study can be found in online repositories. The names of the repository/repositories and accession number(s) can be found in the article/Supplementary material.

## Ethics statement

The animal study was reviewed and approved by the Institutional Animal Care and Use Committee of the College of Animal Science and Technology, Sichuan Agricultural University, Sichuan, China.

## Author contributions

WC, WS, and SL: conceptualization. WC: methodology, resources, data curation, and writing—original draft preparation. SC: software. SC and XJ: validation. WC and WS: formal analysis and investigation. WS, SC, JW, and SL: writing—review and editing. XJ: visualization and supervision. SL: project administration and funding acquisition. All authors have read and agreed to the published version of the manuscript.

## Supplementary material

Supplementary Table 1 All primer sequences. A summary of raw data, clean data, and reads quality of sequenced samples.

Supplementary Table 2 The prediction of known miRNAs and novel miRNAs in each sample.

Supplementary Table 3 The annotation of differently expressed miRNAs between the normal and low-quality semen groups.

Supplementary Table 4 The significantly enriched KEGG pathways of differently expressed miRNAs.

Supplementary Table 5 The significantly enriched GO pathways of differently expressed miRNAs.

Supplementary Table 6 All of the metabolites obtained from the ESI+ and ESI− mode.
